# ‘A priori’ external contextual factors and relationships with process indicators: a mixed methods study of the pre-implementation phase of ‘Communities in Charge of Alcohol’

**DOI:** 10.1186/s12889-022-14411-2

**Published:** 2022-11-29

**Authors:** Elizabeth J. Burns, Suzy C. Hargreaves, Cathy Ure, Susan Hare, Margaret Coffey, Mira Hidajat, Suzanne Audrey, Frank de Vocht, Kate Ardern, Penny A. Cook

**Affiliations:** 1 School of Health and Society, University of Salford, Manchester, UK; 2 Fallowfield Community Guardians c/o School of Health and Society, University of Salford, Manchester, UK; 3 Population Health Sciences, Bristol Medical School, University of Bristol, Bristol, UK

**Keywords:** Alcohol harm, Community health champion, Lay involvement, Pre-implementation context

## Abstract

**Background:**

It is widely recognised that complex public health interventions roll out in distinct phases, within which external contextual factors influence implementation. Less is known about relationships with external contextual factors identified a priori in the pre-implementation phase. We investigated which external contextual factors, prior to the implementation of a community-centred approach to reducing alcohol harm called ‘Communities in Charge of Alcohol’ (CICA), were related to one of the process indicators: numbers of Alcohol Health Champions (AHCs) trained.

**Methods:**

A mixed methods design was used in the pre-implementation phase of CICA. We studied ten geographic communities experiencing both high levels of deprivation and alcohol-related harm in the North West of England. Qualitative secondary data were extracted from pre-implementation meeting notes, recorded two to three months before roll-out. Items were coded into 12 content categories using content analysis. To create a baseline ‘infrastructure score’, the number of external contextual factors documented was counted per area to a maximum score of 12. Descriptive data were collected from training registers detailing training numbers in the first 12 months. The relationship between the baseline infrastructure score, external contextual factors, and the number of AHCs trained was assessed using non-parametric univariable statistics.

**Results:**

There was a positive correlation between baseline infrastructure score and total numbers of AHCs trained (R_s_ = 0.77, *p* = 0.01). Four external contextual factors were associated with significantly higher numbers of lay people recruited and trained: having a health care provider to coordinate the intervention (*p* = 0.02); a pool of other volunteers to recruit from (*p* = 0.02); a contract in place with a commissioned service (*p* = 0.02), and; formal volunteer arrangements (*p* = 0.03).

**Conclusions:**

Data suggest that there were four key components that significantly influenced establishing an Alcohol Health Champion programme in areas experiencing both high levels of deprivation and alcohol-related harm. There is added value of capturing external contextual factors a priori and then testing relationships with process indicators to inform the effective roll-out of complex interventions. Future research could explore a wider range of process indicators and outcomes, incorporating methods to rate individual factors to derive a mean score.

**Trial registration:**

ISRCTN81942890, date of registration 12/09/2017.

## Introduction

Harmful alcohol use accounts for 3 million deaths every year, six deaths every minute worldwide [1] and one in 20 deaths in Europe [2]. Internationally alcohol consumption is recognised as the leading risk factor for deaths and disability among those aged 15–49 years [3]. The global burden of disease, acute injury, and social harm attributable to alcohol use is closely associated with how people drink, how much they drink, as well as where they drink [4]. Fundamentally, alcohol harm worsens inequalities between and within countries [1].

Definitions of hazardous and harmful drinking distinguish differences between consumption levels that increase the risk of harmful consequences and those that are already causing evident harm, before the development of alcohol dependence [5]. In England, prior to the COVID-19 pandemic, some 10.8 million adults were drinking alcohol at hazardous or harmful levels, and a further 1.6 million with mild to severe levels of alcohol dependence [6]. A more recent review of the wide-reaching impact of alcohol misuse found that across the United Kingdom (UK) and Republic of Ireland one in five people were harmed by others’ drinking over the previous year [7]. This has particular importance when countering the persistent argument proposed by the alcohol industry that there is little need for strict alcohol control policies with only a minority of people using their products “irresponsibly” [8, 9].

Long-standing evidence demonstrates how reductions in alcohol harm can be achieved by improving regulation, restricting the availability of alcohol, and increasing opportunities for alcohol screening and brief advice to promote behaviour change [10–12]. Despite rich evidence detailing the effectiveness of such interventions when undertaken by professionals [1, 4, 5, 10–12], it remains largely unknown to what extent lay people can provide a role in alcohol harm reduction efforts. The role of lay people in promoting health and wellbeing has been described in numerous other public health contexts previously as: community health workers; lay health advisors; health champions, or; volunteer ‘support from next door’ [13–15].

Existing evaluations have recognised the importance of a supportive infrastructure when implementing new community health champion programmes [16, 17] but more research is needed to explore what aspects positively influence process indicators such as recruitment, training numbers, and retention. Additionally, there is a large body of evidence highlighting how complex health interventions roll out in identifiable phases or stages [18, 19], with implementation outcomes affected by different external contextual factors at each phase [20–22]. However, research has predominantly been carried out in health and social care systems, rather than community-centred approaches to improve health, wellbeing and the environment, with sustainability research gaining greater attention [16, 21, 23].

Complex public health interventions are characterised by the number of interacting individual and community components with varying degrees of task difficulty, the number of organisations involved in implementation, and the flexibility to tailor interventions to the local context [24]. By better understanding predictors associated with the successful implementation of complex health interventions, the effectiveness of public health programmes could improve. To address these gaps, this study investigated external contextual factors in the pre-implementation phase of an Alcohol Health Champion training programme in the North West of England called Communities in Charge of Alcohol (CICA).

### Aims and objectives

Using a mixed methods approach [25], the aim of this study was to understand the context of roll-out preparation in the pre-implementation phase of the CICA intervention. The key objectives of the study were to:

1. Explore external contextual factors identified by stakeholders a priori to prepare for implementation in each intervention site.
2. Measure numbers of lay people trained as Alcohol Health Champions (AHCs) as a key output indicator in the first 12 months.
3. Test relationships between external contextual factors identified in the pre-implementation phase with numbers of Alcohol Health Champions (AHCs) trained.

## Methods

### The CICA intervention

CICA’s theory of change hypothesised that a two-day Train-the-Trainer event provided to a local CICA coordinator and local lay volunteers would be asset building [26], creating a groundswell of ‘Alcohol Health Champions’ (AHCs) [27]. Training aimed to strengthen skills, knowledge and confidence in evidence-based interventions traditionally used by professionals to reduce alcohol harm, namely: engaging in the local alcohol licensing process to improve the regulation of alcohol, and providing alcohol-related brief advice to promote behaviour change (see Table 1). CICA was designed as a community-based approach to improve health, wellbeing and alcohol risk environments.

**Table 1:** Communities in Charge of Alcohol (CICA) Train-the Trainer Event

**Full day - Day 1: Level 2 Royal Society for Public Health (RSPH) qualification**	
• Alcohol awareness: its effect on health, individuals, society	
• Measuring alcohol consumption and definitions of lower risk drinking	
• How to identify hazardous and harmful drinking including the use of a validated alcohol screening tool such as AUDIT-C	
• Understanding behaviour change and how to offer brief advice	
**Half day - Day 2: Introduction to alcohol licensing (supported by a local licensing officer)**	
• Alcohol licensing awareness – regulation, compliance and enforcement	
• How AHCs can influence alcohol availability and get involved with licensing decisions using existing powers within the Licensing Act 2003	
• How AHCs can establish community action against alcohol harm and access a public licensing register of applications and licences granted where available	
**Half day – Day 2: ‘Train-the-Trainer’ session**	
• Essential information for training preparation and delivery	
• Presentation skills	
• Review and practice	

Train-the-Trainer approaches are designed to enable participants to instruct and support others in subsequent training events they ‘cascade’ [28]. Each CICA coordinator had a target to recruit five AHCs to attend initial training alongside them (so-called ‘first generation’ AHCs), with the expectation that they would cascade training to a further 30 AHCs during the first 12 months. Cascade training was defined as any subsequent CICA training delivered to new recruits by the initial group of AHCs in the 12-month intervention period. The AHC role description explained that volunteers would not have targets for the number of conversations, referrals, or community events they attended; leaving it up to each AHC to decide how (and how often) they used their knowledge and skills in their local community [14]. Local coordinators were not given specific guidance on how to recruit volunteers, other than seeking people who lived or worked in the area, and approaching any existing networks of RSPH Level 1 Health Champions if available.

### Study design

This study focused on the pre-implementation phase of the larger Communities in Charge of Alcohol (CICA) evaluation. CICA was a community-based intervention, evaluated using a quasi-experimental stepped-wedge trial design [27] in ten areas of Greater Manchester (ISRCTN81942890, 12/09/2017). The random order of roll-out for the ten areas was generated by the CICA project’s lead statistician (FdV) using statistical software R, independent from the implementation team as well as process evaluation team.

The methods used in the pre-implementation phase reported here contained three process evaluation components: (1) a qualitative content analysis of external contextual factors identified in preparatory roll-out meeting notes; (2) a descriptive quantitative analysis, counting the number of external contextual factors available in each intervention area prior to roll-out to create an ‘infrastructure score’, and; (3) a quantitative correlation analysis testing relationships between the baseline infrastructure score, individual external contextual factors and the number of AHCs trained.

### Setting

Ten geographic areas were selected by local authority public health teams using a set of available local indicators of alcohol-related harm [29, 30]. All intervention sites contained neighbourhoods that were in the most deprived decile according to the Index of Multiple Deprivation [31] and identified as having high indicators of alcohol harm in comparison to other neighbourhoods within the same local authority.

The CICA intervention launched with Train-the-Trainer events provided by the Royal Society for Public Health (RSPH), with pairs of interventions sites scheduled to roll out in sequence between September 2017 and May 2018. Training was commissioned centrally in the context of the devolved city region of Greater Manchester in the North West of England. Commissioning is a process used by public health leaders in the UK to plan, procure, monitor, and review health improvement, primary prevention, secondary prevention and tertiary prevention programmes [32].

Stakeholders from each area attended preparatory meetings during the two to three months leading up to the roll-out of the intervention, marked by the area’s dedicated Train-the-Trainer event. Preparatory roll-out meetings were hosted using audio teleconferencing and convened by an overall programme manager supporting the coordinated implementation of CICA. Local stakeholders included commissioning leads, alcohol licensing leads and local service providers from the ten programme areas. The research team were invited to observe the meetings and share details of the process evaluation.

### Data collection

#### Preparatory roll-out meeting notes

Preparatory roll-out meeting notes were shared with the research team as secondary data. Notes were recorded for each pair of areas between July 2017 and April 2018, structured around a roll-out checklist created by stakeholders to guide preparation for each Train-the-Trainer event.

#### Licensing authority webpages

One of the data items raised in the preparatory meetings was the availability of an online public licensing register. Since this was not confirmed within the preparatory meetings, it was checked a priori. Each local licensing authority’s webpage was used to identify the availability of an online public licensing register. All licensing authorities in England and Wales are obligated to provide public access to contents of their licensing register [33, 34]. When fully comprehensive, these registers contain details of new alcohol licensing applications as well as copies of existing premises licences issued. Full public access was defined as an online public register containing details of the named Designated Premises Supervisor, Opening Times, Permitted Activities and Hours Granted, and Conditions attached to the licence. The research team (EJB and SCH) recorded, a priori, which licensing authorities provided full access.

#### Process indicator: numbers of alcohol health champions trained

Training registers provided data on the numbers of AHCs trained within the first 12 months of the intervention. Since areas had different start dates (due to the stepped roll-out), the total data collection period was 20 months (September 2017 and April 2019). These data differentiated between Train-the-Trainer event attendance (first generation AHCs), cascade training attendance (second generation AHCs) as well as how many lay people participated in the training compared to professionals. The purpose here was to assess the reach of recruitment efforts to lay people within the community. A professional was defined as a person in paid employment with existing specialised knowledge of alcohol, substance misuse and/or public health practice who attended the training as part of their responsibilities for the CICA intervention area.

### Analysis

The first step in content analysis involves the unit of analysis to be selected. Then, following a comprehensive review of the collected data, information relevant to the research question is identified [35]. The unit of analysis selected was drawn from the description of ‘tasks per roll-out’ as documented in secondary data meeting notes. A summative, deductive approach was taken whereby the keywords on the roll-out meeting checklist were organised into a categorisation matrix, identifying content that described external contextual factors. While summative approaches to content analysis can include latent analysis to define the underlying meaning of the text, here descriptions of categories stayed close to the text [36].

As employed by other studies [25, 37], one additional step was taken to count the number of factors per area. Counting how many external contextual factors were in place prior to CICA’s roll-out generated a total baseline ‘CICA infrastructure’ score for each area to a maximum total of 12. Similar to other studies using content analysis [38], only one item per area was counted if recorded as available or in place in the pre-implementation phase. The ‘CICA infrastructure score’ enabled comparisons to be made between areas.

CICA coordinators who had been involved in the roll-out meetings were given the opportunity to review the matrix of external contextual factors and infrastructure scores calculated for their individual area at a follow-up interview. Such member-checks can enable participants to feed back to researchers whether findings are true to their experiences [39]. These interviews took place nine to 12 months after the first training event and were part of a wider qualitative study into local coordinators’ perspectives of barriers and facilitators affecting sustainability, findings of which will be published elsewhere.

A Spearman’s rank correlation test measured the association between the infrastructure score and the number of AHCs trained. This was followed by Mann Whitney U-tests performed on each individual external contextual factor. Analyses were carried out using IBM SPSS Statistics 25.

## Findings

Twelve categories of external contextual factors were identified within roll-out meeting notes in the pre-implementation phase (see Table 2). Counting the number of external contextual factors generated a total baseline ‘CICA infrastructure’ score for each area to a maximum total of 12.

**Table 2:** Summary of external contextual factors organised into categorisation matrix

Keywords from roll-out meeting checklist	Categories of external contextual factors
Confirm champions and invite	1. Healthcare provider in place to coordinate the intervention
	2. Contract in place with a commissioned service
	3. Staff stability with staff in post at outset
	4. Existing pool of RSPH Level 1 Health Champions to recruit from
	5. Pool of other volunteers to recruit from
	6. Formality of volunteer arrangements
Confirm RSPH centre status	7. RSPH training centre status affiliated
RSPH Qualification	8. Local CICA coordinator registered trainer with RSPH
Confirm Public Health/Other Input	9. Support from local Director of Public Health through the allocation of resources
	10. Evident support from elsewhere in the local authority e.g. elected members
Confirm licensing input	11. Support from a licensing officer from the local licensing system
Licensing information: register of premises	12. Public licensing register in place

### CICA training response

Within the 12-month intervention period, a total of 123 participants were trained to become AHCs with 77% (*n* = 95) identified as lay people living or working within the community and 23% attending in paid professional roles (*n* = 28). Nine out of ten areas trained at least some AHCs. Of the nine areas, seven completed the full 12-month intervention period, defined as successfully delivering at least one cascade training event. Two areas discontinued CICA work after six and 9 months respectively.

Variation between the areas was noted in the number of months between first generation training of AHCs (Train-the-Trainer events) and second-generation training (cascade training events) ranging from two to 12 months. Table 3 shows that out of seven areas, most CICA intervention sites that ran for the full year delivered at least one cascade event, with five delivering one cascade event per area and one area delivering four cascade events. The randomly assigned area numbers used for reporting in Table 3 are consistent with previous publications on this intervention.

**Table 3:** Timeline of cascade training events, total AHCs trained and infrastructure score

CICA area	Month number (number of months after initial training)				Total AHCs trained	Total lay people trained^a^	Infrastructure score^a^
	Cascade 1	Cascade 2	Cascade 3	Cascade 4			
Area 1	Month 6	–	–	–	16	14	10
Area 2	Month 3	–	–	–	20	17	9
Area 3	Did not cascade – withdrew Month 6				13	8	5
Area 4	Month 12	–	–	–	11	7	10
Area 5	Withdrew at baseline				0	0	0
Area 6	Month 10	–	–	–	9	8	7
Area 7	Did not cascade – withdrew Month 9				7	5	4
Area 8	Month 2	Month 4	Month 9	Month 10	22	17	10
Area 9	Month 7	Month 10	–	–	15	13	7
Area 10	Month 8	–	–	–	10	6	4

^a^Spearman’s rank correlation coefficient r_s_ = 0.77, *p* = 0.01

At follow-up interview, CICA coordinators from seven out of ten areas reviewed these initial findings. Six interviews took place at the 12-month follow-up period and one after 9 months when the area withdrew from the project earlier than planned. In six areas, no amendments were made and in one area, one item was amended following further clarification. Of the three areas that could not be member-checked by a CICA coordinator, two CICA coordinators were lost at the follow-up stage (6 months and 12 months) and the third area did not roll-out and withdrew from the programme.

### Infrastructure scores and relationship with numbers trained per area

A positive correlation was found between an area’s total infrastructure score and the total numbers of lay AHCs trained in the first year (Table 3; *r*_*s*_ = 0.77, *p* = 0.01). Mann-Whitney U tests were performed on each external contextual factor in relation to the numbers of lay AHCs trained (see Table 4). From this, four external contextual factors were found to be correlated with higher numbers of lay people trained: having a healthcare provider in place at the outset to coordinate the intervention (*p* = 0.02); a contract in place with a commissioned service (*p* = 0.02); a pool of other volunteers to recruit from (*p* = 0.02); and, formal volunteer arrangements (*p* = 0.03).

**Table 4:** Comparison of associations between external contextual factors and training

External contextual factor content category	Number of areas with characteristic	Mann-Whitney U test statistic	Significance
Healthcare provider in place to coordinate the intervention	7	21	0.02
Contract in place with a commissioned service	6	22.5	0.02
Staff stability with staff in post at the outset	9	9	0.20
Pool of other volunteers to recruit from	7	21	0.02
Existing pool of RSPH Level 1 Health Champions to recruit from	0	–	
Formality of volunteer arrangements	5	23	0.03
RSPH training centre status affiliated	5	19	0.22
Local CICA coordinator registered trainer with RSPH	3	17	0.18
Support from local Director of Public Health through the allocation of resources	9	9	0.20
Evident support from elsewhere in the local authority	3	17	0.18
Support from a licensing officer from the local alcohol licensing system	8	8.5	1.00
Public licensing register in place at the outset	4	13.5	0.76

## Discussion

The aim of this study was to understand the context of roll-out preparation, in the pre-implementation phase of the CICA intervention. Existing research highlights the importance of having a supportive infrastructure when implementing health champion programmes. However, there is limited evidence around possible relationships between individual external contextual factors and process indicators. Here we found that areas with more infrastructure in place in the pre-implementation phase trained more AHCs in the first 12 months, with four key enabling factors.

Two enabling external contextual factors centred around ‘commissioner-provider’ relationships: having a named healthcare provider in place who would lead and coordinate the intervention, and delivery specified within an existing contract with local commissioners. Consistent with findings reviewed elsewhere [16], a ‘supportive infrastructure’ includes an organisational model that has a coordinating function. Allocating dedicated time, resources and support for staff involved in community engagement initiatives requires sustainable investment (14–17). While this study found better-performing areas had the delivery of CICA specified within an existing contract, the pre-implementation phase did not include a review of the exact components of those contracts.

The other two factors revolved around the volunteer capacity within communities. Higher numbers of AHCs were trained when there were existing pools of volunteers to recruit from, and formal induction arrangements were in place such as an induction to volunteering and indemnity cover from the healthcare provider. This is of interest, since bureaucracy or feeling a burden of responsibility have been previously found to be potential barriers to engagement for some community champions rather than an enabler [16, 40]. Analysis of interviews with AHCs reported in a sister paper [41] suggested that the AHCs valued being part of an organised structure and required ongoing support from the local coordinator.

Importantly, this study had a longitudinal design. Focusing on the pre-implementation phase preceding the roll-out of the intervention, data were collected a priori (two to three months prior to roll-out), while numbers of trained individuals were collated 12 months after roll-out. One of the benefits of a longitudinal design is the prospective data collection method as it minimises the effects of recall bias [42]. A recent UK rapid scoping review highlighted the value of having comparative elements when investigating community health champion interventions and testing indicators that could be used at a local level when monitoring a programme’s impact over time [16].

The original aim had been to train 350 AHCs across the 10 areas. As the first alcohol-focused champion role of its kind, it is unknown whether the topic of ‘taking charge of alcohol’ in CICA was a less attractive volunteering role compared to other health domains previously researched [43]. It is also possible that action to reduce alcohol harm was perceived as a professional-determined priority, rather than what mattered most to the community [44]. A sense of community or feeling part of a community have also been identified as important to the implementation of a champion role [15, 44, 45]. However, community insights were not explored in the pre-implementation phase so this cannot be assumed.

Commissioner-provider relationships within the healthcare system have long been identified as key to effective change [46] but are highly influenced by local contextual conditions [47]. In England, between 2015 and 2019, local authority alcohol harm prevention budgets were cut from 35 to 15% and public health grants reduced by £700 million [48, 49], which may account for the challenges documented in different areas. There is also international recognition of a lack of investment to finance alcohol prevention programmes [1].

These findings could aid decision-makers in the UK and globally to prioritise investment in infrastructure prior to the launch of a complex intervention to reduce alcohol harm. Investment for an intervention such as CICA needs to include building volunteer capacity among non-volunteers, especially where existing pools of volunteers are not established, and consider how adopting formal practice from human resources could influence volunteer outcomes [50]. While volunteer passporting schemes could offer a coordinated approach for formalising volunteering arrangements, future research could explore the components that minimise bureaucracy for community members. Separate work would still be needed to build community volunteering capacity where it does not exist [51] and allowing adequate preparation time and resources for this [52].

There is an important need for evidence for those planning community action on alcohol considering recent forecasts predicting that the global target to reduce harmful use of alcohol by 10% by 2025 [10] is on a poor trajectory [53, 54]. These concerns are exacerbated by more recent evidence of increasing use of alcohol during the COVID-19 pandemic [55, 56] and alcohol related harm, with fewer opportunities for early intervention and treatment [57]. If the full impact of evidence-based population health interventions is to be achieved, and in novel ways, barriers and facilitators that exist outside the boundaries of the intervention itself should continue to be investigated, not only to assess a community’s ‘readiness’ to roll-out but also to inform how research should proceed between phases [58, 59]. Assessing infrastructure using a recognised assessment tool could also strengthen comparability of study findings [19, 60].

### Strengths and limitations of the study

This study contributes to the understanding of infrastructure in the pre-implementation phase of a complex public health intervention to reduce alcohol harm. Since infrastructure data were recorded prior to the start of the intervention, the assessment was not influenced by the knowledge of the final numbers trained. In CICA, the definition of the community was a ‘place’ that had been selected based on available alcohol harm data and within an English context. While the transferability of these findings may therefore be limited, due to the global issue of alcohol harm, the findings could be of benefit to any area setting up a community approach such as CICA.

In this study there is a recognised limitation of using an indirect observation method such as content analysis, as it does not account for potentially differing contributions of key informants to preparation meetings [39]. Additionally, it is recognised that member-checks as a basis for verification of overall results should be treated with caution since participants may not be able to recognise particular experiences and responsive investigators may inadvertently constrain their results [61, 62]. This study also did not include an opportunity for key informants to rank or rate each external contextual factor to derive a mean score [19, 22, 63]. Additionally, only 12 contextual areas were considered a priori. As found in Ure et al. [64, 65], in-depth follow-up interviews with stakeholders identified a wider range of enabling factors as well as barriers that influenced recruitment and numbers trained.

## Conclusion and recommendations

The success of future health champion-based interventions may be optimised if implementation leaders identify a minimum set of external contextual factors to invest in and establish during the pre-implementation phase. Findings suggest that a roll-out checklist of tasks would benefit from co-production to gain community insights into external contextual factors, informed by a theoretical framework to guide its development and inform decisions as to whether the community is ready to roll-out.

In CICA, four external contextual factors were associated with the successful recruitment and training of lay people in an alcohol focused health champion role. Future research could incorporate quality rating external contextual factors and investigate the degree to which they impact on a wider range of process indicators as well as the intervention’s outcomes, further refining best practice for the pre-implementation phase.

## Data Availability

The datasets analysed during the current study are not publicly available as participants did not agree for the data to be shared publicly but are available from the corresponding author on reasonable request.

## Abbreviations

AHC: Alcohol Health Champion
AUDIT-C: Alcohol Use Disorder Identification Test-Consumption
CICA: Communities in Charge of Alcohol
RSPH: Royal Society for Public Health
